# Intelligent Control System for the Hard X-Ray Nanoprobe Beamline Beam Optimization Based on Automatic Evolution Algorithm and Expert System

**DOI:** 10.3390/s24227211

**Published:** 2024-11-11

**Authors:** Yuhao Liu, Ying Zhao, Yan He, Zhaohong Zhang, Aiguo Li

**Affiliations:** 1 School of Microelectronics, Shanghai University, Shanghai 200444, China; liuyuhao@sari.ac.cn; 2 Shanghai Synchrotron Radiation Facility, Shanghai Advanced Research Institute, Chinese Academy of Sciences, Shanghai 201210, China

**Keywords:** expert system, automatic evolutionary algorithm, SSRF, BL13U, beamline control

## Abstract

A synchrotron radiation beamline automatic optimization system has been used in the Shanghai Synchrotron Radiation Facility, improving the optimization efficiency, but it does not store and use the beamline adjusting experience, and cannot quickly optimize and store the experienced improvement. The expert system combined with an automatic evolutionary algorithm is used for intelligent beamline optimization; the algorithm initialization is optimized by invoking database experience, the convergence is quickly completed near the optimal solution, and the system’s learning is improved by storing experience results. The software was designed on the EPICS (Version 3.15) platform, which was used to implement the algorithm in Python language, the expert database was developed with MongoDB tool (Version 4.0.27), and the upper application interface was designed with CSS software (Phoebus Version 4.7.2). The system was successfully tested on the BL13U hard X-ray nanoprobe beamline of Shanghai Synchrotron Radiation Facility. The results show that the maximum convergence time of a single objective with four-axis degrees of freedom is about 2 min, and the speed is increased by 15 times. The solution set obtained by using multi-objective two and four-axis degrees of freedom is better overall. The system can effectively improve the optimization efficiency and effect, and its universality can be extended to other synchrotron radiation devices and beamlines to promote the development of intelligent beamline modulation technology.

## 1. Introduction

Synchrotron radiation nanoprobe technology can deeply probe the internal structure of the study object without damaging the sample, and has high detection sensitivity and high transmission, providing a powerful tool for the research of physics, biology, semiconductors and materials [1,2,3]. The Shanghai Synchrotron Radiation Facility (SSRF) hard X-ray nanoprobe beamline is equipped with a collimator (FMB, Oxford), a multilayer monochromator (FMB, Oxford), a 10 nm focusing system (Customized in China) and other important optical components; its X-ray focusing performance can reach 10 nm ultra-high spatial resolution and 2 nm scanning motion repetition accuracy, provided by nanofilm fluorescence imaging, nanofilm absorption imaging, nanofilm diffraction and coherent diffraction imaging, and other experimental methods [4]. It is one of the most effective means to help scientists carry out cutting-edge scientific research on the nano scale. The excellent experimental performance determines the large number and complex structure of beamline optical components, and the necessary conditions for regulating high-quality light to carry out a series of experiments, but the factors influencing beam of light quality are numerous and difficult to define, and cannot be solved by mathematical equation modeling methods.

The flux and spot positions of hard X-ray nanoprobe beamlines are necessary conditions enabling users to conduct experiments. The manual optimization requires experienced scientists to adjust the motor of the slit, collimator (FMB, Oxford), monochromator (FMB, Oxford), focusing mirror (Axilon, Germany) and other optical elements one by one to adjust the optical attitude of the elements until the quality of the beam meets the experimental requirements. Manual alignment can only adjust and optimize the feedback of one single motion axis of the optical element, resulting in the problem that the subset of the global solution space is extremely large or even impossible to exhaust. Moreover, the whole process is heavy in terms of workload, low in efficiency, time-consuming and laborious, wasting precious beam time. These requirements and challenges have prompted the study of beam optimization methods based on the automatic beamline alignment strategy.

In the 1990s, Roberto Poboni and Roberto Pugliese et al. established an adaptive control system at Super-ESCA beamlines based on the analysis and utilization of the basic knowledge of fuzzy logic. The FuzzyCLIPS 1 tool, shell6 extended version based on CLIPS rules, is developed to represent and manipulate fuzzy facts and rules, and the control model of the Super-ESCA beamline system is simplified, such that FuzzyCLIPS can apply the fuzzy logic control strategy to the optimization of the beamline [5]. Subsequently, Roberto Pugliese proposed the concept of an intelligent system based on the previous method to improve the optimization efficiency of the beam. Based on the intelligent system, the focusing task of the whole beamline is transformed into the optimization work of a single optical element, which further improves the degree of automatic optimization. The method was applied to the BM14 site of the European Synchrotron Radiation Facility (ESRF) [6]. In 2001, a research team led by Olivier Hignette developed a new optimization method for ESRF’s Kirkpatrick–Baez (K-B) reflecting mirror system using wavefront analysis. This method automatically processes the wavefront information by the sequential scanning of the slit and obtaining the position information of the device in the focal plane, and then realizes the automatic adjustment of the alignment and bending operation of the KB mirror. The projection phase image of the submicron structure of the KB mirror system can be obtained by this method, and the spot can be accurately predicted [7]. However, the above several beam automatic alignment methods could hardly reach the degree of intelligent beam modulation, until 2015, when Singapore Synchrotron Light Source (SSLS) researchers proposed to apply the Genetic Algorithm (GA) for automatic beamline optimization. The auto-tuning optimization of the beam line is realized by the global optimization genetic algorithm. A program based on GA was tested at the XAFCA beamline of the Singapore Synchrotron Light source. It was demonstrated that the method can optimize all the optical components of the XAFCA beamline [8]. In 2017, in order to further improve the speed of algorithm optimization, SSLS researchers proposed to adjust and modify the architecture of the algorithm, the GA and Differential Evolution (DE) algorithm was used for automatic optimization, and the strategy Observer Mode for Evolutionary Algorithm (OMEA) was used. The graphical interface of AI-BL 1.0 is further developed based on Labview software, which gives users an efficient way to customize the algorithm [9]. The automatic beam alignment control system established by SSLS is developed based on LabVIEW. However, most synchrotron radiation light sources in the world implement their control software based on Experimental Physics and Industrial Control System (EPCIS) [10] and Tango [11], and the applications developed based on LabView have weak portability and universality. Therefore, it is difficult to extend the above intelligent optimization method to other synchrotron radiation light sources. In 2020, scientists from Shanghai Synchrotron Radiation Facility (SSRF) developed an automatic beam optimization system based on EPICS and DE, and carried out online tests on the BL14B1 beamline, obtaining a result wherein the flux size of a single objective converged to the global optimal solution. This provides a good research basis for the intelligent beam alignment method to be transplanted to other synchrotron radiation facilities [12]. Subsequently, the researchers at SSRF further designed and developed a multi-objective automatic beam alignment method based on spot stability and flux. The method modified the evolutionary algorithm, replaced DE with multi-objective optimization Non-dominated Sorting Genetic Algorithm II (NSGA-II), and realized the evolutionary algorithm through Python language [13]. Recently, scientists optimized the flux and spot size with different evolutionary algorithms (EA) and swarm intelligence (SI) algorithms including GA, NSGA-II, Particle Swarm Optimization (PSO) and Artificial Bee Colony (ABC). The results show that some algorithms and techniques may give better performance than others for every optimization problem. In this study, a program for automatic online beamline optimization using NSGA-II is presented [14]. There were also simulation tests performed specifically for multi-objective GA, and the results also show that the algorithm and technique were effective and could improve the efficiency of beamline optimization [15]. In accelerator physics, the application of machine learning and nature-inspired optimization methods, including GA and PSO, can be found [16,17,18]. In addition, the combination of beam optimization and experimental data acquisition, even without the use of advanced algorithms such as GA, NSGA-II and PSO, can greatly improve the automation and intelligence of the beamline [19,20].

Previous beam optimization methods based on automatic beamline alignment strategies have successfully solved many of the problems of manual beamline alignment, but these systems did not take advantage of scientists’ previous beamline alignment experience. They often repeat the alignment process under similar beamline conditions and cannot store the experience of adaptive learning and improvement, so efficiency is affected. Hard X-ray nanoprobe beamline has been left open to users, and so a lot of manual beam optimization experiences and data are available. In this paper, the expert system [21,22,23,24] in artificial intelligence technology was applied to the beam optimization of the hard X-ray nanoprobe beamline, and the intelligent controller was designed in combination with the automatic evolutionary algorithm, while the beam quality feedback mechanism was used to construct the transfer function of the control system, aiming at establishing an efficient intelligent beamline optimization control system. The DE algorithm was run for a single objective—maximum flux. The NSGA-II algorithm was run for both objectives—maximum spot stability and maximum flux. Each time the beamline was optimized, the system matched the experience according to the requirements, called the expert system database, and transmitted the matrix vector information containing the optimal optical attitude of each component to the algorithm to achieve the initial optimization. After the fast convergence was completed, the results were stored in the expert database to ensure the adaptive learning of the system.

The EPICS control platform(Version 3.15) is designed as the system software framework to realize intelligent optimization, the program of the automatic evolution algorithm was realized by use of the Python programming language, the expert database was designed by the MongoDB tool l(Version 4.0.27), and the upper application interface was developed in the Control System Studio (CSS) (Phoebus Version 4.7.2) software [25]. The PyMongo(Version 4.0.27) [26] interface was developed to realize the transfer of call and storage instructions between the program and the database, and the PyEpics interface [27] was developed to realize the transmission of control signals between the program and the EPICS control platform. In this process, the linkage of the drive motor was generated to optimize the optical attitude of the component to achieve beamline modulation. A self-learning intelligent beam-tuning system with optimal initialization, fast convergence and effective storage is realized.

The designed system was applied to the BL13U beamline equipment, and several rounds of tests were completed, with the expected goal being achieved. The effectiveness and robustness of the system are verified, and the universality of the system can be extended to many similar synchrotron radiation devices and beamlines, which provides a reference for intelligent beamline modulation.

## 2. Materials and Methods

### 2.1. Overall System Architecture

**Figure 1:**
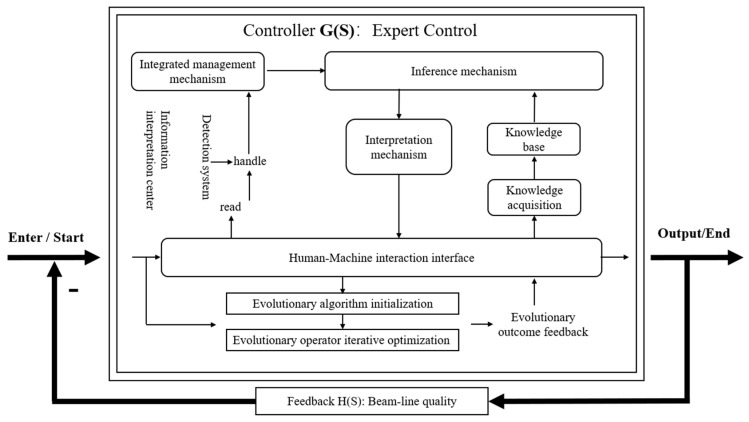
Overall framework of the intelligent control system for beam optimization.

The overall framework of the intelligent control system for beamline optimization is shown in Figure 1, including the controller and feedback.

In the system framework, the automatic evolutionary algorithm combines with the expert system to generate an intelligent controller, G(S). Simultaneously, the feedback function H(S) is established based on real-time beam performance, forming a closed-loop transfer function. This configuration allows for the efficient initiation and termination of beam regulation, thereby achieving highly intelligent beam control.

### 2.2. Intelligent Controller Design

**Figure 2:**
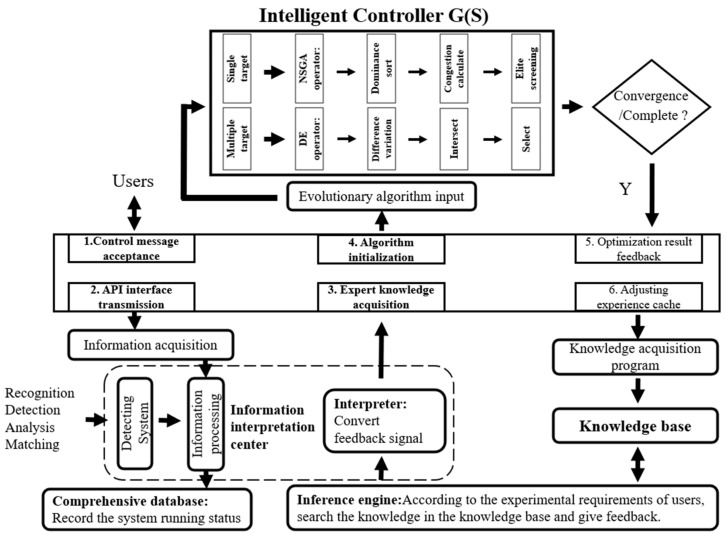
Intelligent controller architecture.

The detailed structure of the intelligent controller within the system is shown in Figure 2. The construction method of the intelligent control system for beam optimization involves establishing expert control based on the intelligent controller, G(S), as the forward channel function in the closed-loop optimization transfer function model. The feedback function, H(S), is formed by utilizing feedback spot position and parameters such as flux intensity as the fitness function. This feedback function, H(S), serves as the reverse channel function in the architecture of the closed-loop optimization transfer function. The whole process of intelligent beam tuning of the system is shown by the arrow in Figure 2, starting from the client and ending in the sequence of operation 1 to operation 6. By operating within the dotted lines in the diagram, the user’s experimental requests are identified, analyzed, and matched, and converted into experiences that can be fed back to the algorithm for initialization.

The intelligent controller comprises an expert system and a differential evolution algorithm. The comprehensive knowledge base of the expert system offers an initial population that is close to the optimal solution for the global evolutionary algorithm. The automatic evolutionary algorithm then optimizes the motor based on this foundation, ensuring that the optimal individuals in the population converge. As a result, all motion axes of the entire beamline attain the optimal state.

The operation process of the controller architecture is as follows: (1) The user interacts with the interface based on the beam-adjusting requirements for the desired experiments. They select the optical components that need adjustment, which vary depending on the specific experiment at the nanoprobe beamline. This selection becomes the instruction for the beam-adjusting operation and is entered into the system. (2) The instructions are transmitted to the information interpretation center for comprehensive processing. This process converts human behavior into machine language, which is then analyzed, identified, detected, and matched. After processing, the information is sent to the inference machine of the controller through the comprehensive database. (3) Upon receiving the information, the inference machine sends a request instruction to the knowledge base of the controller. It retrieves beam adjusting experiences from the knowledge base using specific access channels and provides corresponding expert advice to the information interpretation center. (4) The interpreter in the information interpretation center processes and converts the experience, transforming machine language into human-readable descriptions. This feedback is then provided to the interactive interface of the controller. (5) The interactive interface transmits instructions to the program through a designated communication interface to initiate initialization. The system program performs optimization operations based on the specified criteria for beam performance improvement. It adjusts the appropriate optical components to achieve the best alignment under the current conditions, records the beam adjusting process, and ultimately provides feedback to the user. (6) The feedback results are received by the knowledge acquisition program and incorporated into the knowledge base through the management mechanism. This helps enhance and expand the expert experience contained within the knowledge base, enabling the system to self-adapt and upgrade.

### 2.3. Construction of Beamline Optimization Model

**Figure 3:**
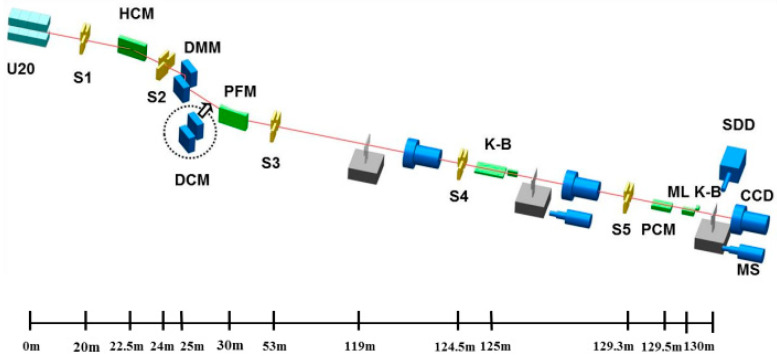
Layout diagram of optical components in a hard X-ray nanoprobebeamline.

The layout diagram showcasing the optical components of the hard X-ray nanoprobe beamline is shown in Figure 3. To achieve the best beam performance as it passes through each optical component, the precise position/attitude of each component needs to be determined for beam optimization. However, since the beamline operates as a high-precision motion system, the relationship between beam performance and optical components is quite intricate and cannot be explicitly modeled. Hence, this paper establishes an optimization model using the automatic evolutionary algorithm for the three key elements of the problem: decision variables, objective functions, and constraint conditions.

The change in attitude depends on the interconnected behavior of multiple motors within the component. Consequently, the moving motor is considered as the decision variable in the experimental setup, and modifying the position attribute of the decision variable affects the optical attitude of the optical component. Beam of light performance serves as the objective function when constructing the optimization model, playing a crucial role in evaluating the effectiveness of decision variables for optimization. Additionally, the range of motion for each motor shaft corresponds to the constraint condition for the optical devices.

The beamline optimization with a single objective is described by an abstract function of Formula (1). In this function, the variable x belongs to the set of real numbers D, and f represents the objective function associated with the custom variable x.

(1) $${f(x}^{*})=\max\left(f\left(x\right)\right),\;x\in D$$

In this paper, the differential evolution algorithm is chosen as the approach for seeking decision variable data in the optimization and regulation problem of a single-objective beam. The optimization model for the single-objective beam is presented as follows:

(a) The motor motion axis corresponding to each optical component in the beamline is quantified by a decision, and is presented in the form of a dimension vector as shown in Formula (2).
  (2) $$P_{i}\left(g\right)=\left[P_{i,j}\left(g\right)\right],\;(i=1,2,\ldots ,n;j=1,2,\ldots ,d)$$
(b) The initial position information of the motor motion shaft is generated using random computer simulation, which in turn determines the optical attitude of the initial optical component, as illustrated in Formula (3).
  (3) $$\left[P_{i,j}\left(0\right)\right]=rand\left[0,1\right]*\left(P_{j}^{max}-P_{j}^{min}\right)+P_{j}^{min}$$
(c) A “mutated” beamline state is generated through the differential operation between beamline states, as demonstrated in Formula (4).
  (4) $$V_{i}\left(g\right)=P_{n_{1}}\left(g\right)+F*\left(P_{n_{2}}\left(g\right)-P_{n_{3}}\left(g\right)\right)$$
(d) The replacement of dimensional vectors between the old and new beamline states results in the generation of the “test” beamline state, as illustrated in Formula (5).
  (5) $$U_{i,j}\left(g\right)=\left\{\begin{array}{c} V_{i,j}\left(g\right),\;if\;rand\left[0,1\right]<cr\\ P_{i,j}\left(g\right),\;else \end{array}\right.$$
(e) Compare beam of light fitness, as shown in Formula (6).
  (6) $$P_{i}\left(g+1\right)=\left\{\begin{array}{c} U_{i}\left(g\right),\;f\left(U_{i}\left(g\right)\right)>f\left(P_{i}\left(g\right)\right)\\ P_{i}\left(g\right),\;else \end{array}\right.$$
(f) Repeat (a) to (e) on the basis of the results in (e) until the end condition is satisfied to obtain the best optical attitude, as shown in Formula (7).
  (7) $$P_{i_{END}}\left(g\right)=\left[P_{i,j}\left(g\right)\right],(i=1,2,\ldots ,n,j=1,2,\ldots ,d)$$

Through the model, the attitude of each succeeding generation of optical devices improves compared to the previous generation. This progress continues until the optimal value of the decision variable is found within the solution space, resulting in the attainment of the best beam performance.

Similar to the single-objective beamline optimization problem, the objective function f(x) = −φ(x) can convert the objective of optimized design, max φ(x), into min f(x). Similarly, in the multi-objective beamline optimization problem with M design objectives, they can be converted to min f_i_(x)(I = 1,2,…,M). If we consider the M objective functions f_i_(x) as M components of the vector objective function f(x), then the expression for multi-objective beam optimization shown in Equation (8) can be obtained.

(8) $$F(x)=min\;f\left(x\right)=min{\left(f_{1}\left(x\right),f_{2}\left(x\right),\ldots ,f_{M}\left(x\right)\right)}^{T}x\in R^{n}$$

$$s.t.g_{i}(x)\leq 0,i=1,2,...,q$$

$$h_{j}(x)\leq 0,j=1,2,...,p<n$$

Refer to the feasible region: $D=\left\{x\right|h_{j}\left(x\right)=0,j=1,\ldots ,p,g_{i}\left(x\right)\leq 0,i=1,2,...,q,x\in R^{n}\}$ This means minimizing the vector objective function f(x) within the feasible domain D.

The model consists of several nonlinear sub-objective functions. During the optimization process of decision variables, simultaneous directional optimization is conducted for these functions. This compromises the optimization effect of multiple sub-objective functions, following the concept of multi-index comprehensive optimal optimization for beam of light performance.

**Figure 4:**
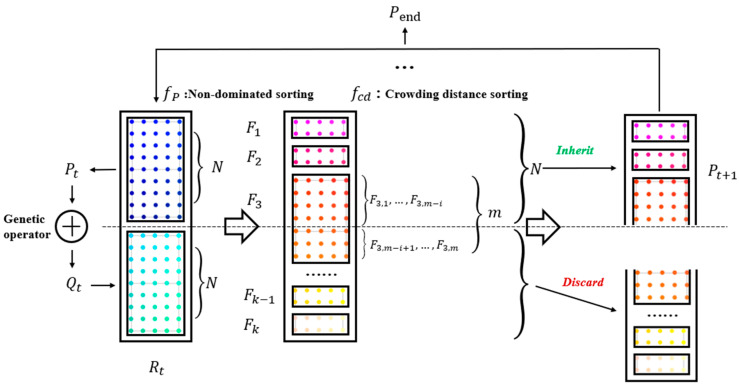
Solution of the multi-objective beamline optimization model based on the NSGA-II algorithm.

Figure 4 depicts the solution diagram of the multi-objective beamline optimization model based on the NSGA-II algorithm. The solution method is described as follows:

(a) The position information of the motor, which includes the optical posture within the optical component, is initialized as the initial population $P_{t}$ with a total number of individuals N. Then, with the current initial parent population $P_{t}$, a variant offspring $Q_{t}$ with an equal number of individuals is generated using the evolution operator. The aforementioned process is illustrated in Equation (9).
  (9) $$Q_{t}=f_{DE}(P_{t})$$
  The child $Q_{t}$ corresponds to the “variant” optical attitude.
(b) The parent population $P_{t}$ and the child population $Q_{t}$ are merged to form a new parent population $R_{t}$, as illustrated in Equation (10).
  (10) $$R_{t}={\left(P_{t},Q_{t}\right)}^{T}$$
(c) At this point, the population $R_{t}$ contains 2N individuals, and $R_{t}$ is efficiently sorted into non-dominated categories to obtain various Pareto sets that differentiate the quality degree of the optical pose, as demonstrated in Equation (11).
  (11) $$\left(F_{1},F_{2},F_{3},\ldots ,F_{k-1},F_{k}\right)=f_{P}(R_{t})$$
(d) After completing the non-dominant ranking, the optical pose individuals from each rank are sequentially added to the next generation population $P_{t+1}$, following the rank order of optical pose quality. This process continues until the number of individuals in the Pareto set of the current rank exceeds the initial population size N, as depicted in Figure 4. Since the optical pose quality level is 3 and the set with m individuals cannot all be accommodated, a crowding ranking operation is performed on the collection of individuals at the $F_{3}$ level. Individuals with greater crowding are then selected and successively added to $P_{t+1}$ until the number of individuals in $P_{t+1}$ reaches N. The crowding ranking operation is described in Equation (12).
  (12) $$\left(F_{3,1},F_{3,2},\ldots ,F_{3,m-i}\right)=f_{Cd}(F_{3})$$
  $$P_{t+1}\leftarrow (F_{1},F_{2},(F_{3,1},F_{3,2},\ldots ,F_{3,m-i}))$$
  Among i∈[0,m], the remaining solutions are discarded.
(e) Repeat steps (a) to (d) based on the results from step (d) until the termination condition is met, in order to obtain the solution set that contains the best non-dominant Pareto optical attitude, as illustrated in Equation (13).
  (13) $$F_{best}=f_{P}(P_{end})$$

Through the repeated operation of evolve-fast non-dominated sort and crowding sort in the solution space corresponding to the decision variable (travel range), the optical attitude of each generation improves compared to the previous generation. This process continues until the best optical attitude Pareto front is discovered in the solution space, ultimately leading to the identification of the equivalent solution set that contains the best beam of light performance.

### 2.4. Expert System Design

**Figure 5:**
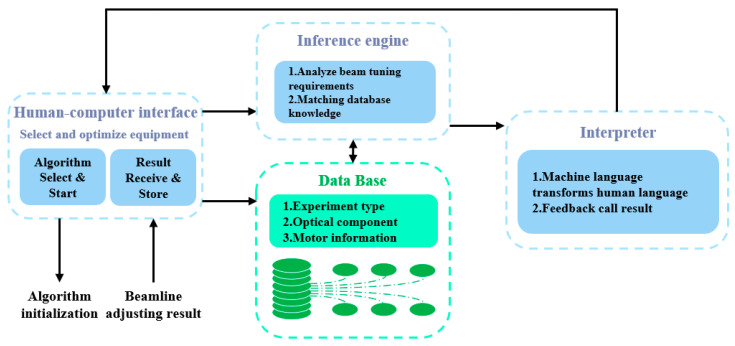
Expert system for beamline modulation control, including human–machine interface, interpreter, inference machine and database.

The structure of the expert system for beamline modulation control is shown in Figure 5. The upper layer interface is the interface for interaction between the user and the system. The user selects the optimized object according to the specific experimental requirements and sends commands and signals to the system. The interpreter decodes the knowledge containing the beamline adjusting experience, converts it into human language for feedback and description, feeds back the corresponding call results according to the user’s experimental requirements, and completes the initialization work based on these results. The algorithm quickly evolves to a convergent state and stores the results in the database to achieve system improvement.

### 2.5. Software Design and Implementation

The intelligent control system utilizes the EPICS system platform as the foundation for implementing an intelligent optimization system. EPICS is a distributed control system developed collaboratively by the Los Alamos National Laboratory and the Argonne National Laboratory in the United States. It is widely employed in numerous large-scale scientific research projects globally. EPICS offers stability, flexibility in system architecture, openness, scalability, as well as a plethora of freely available tools and technical support.

**Figure 6:**
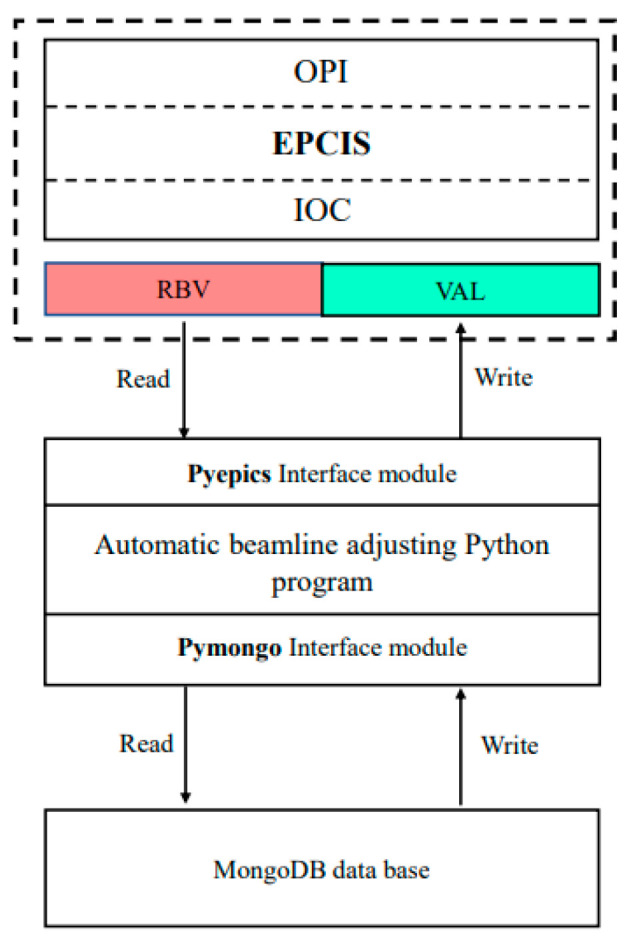
System software structure design.

**Figure 7:**
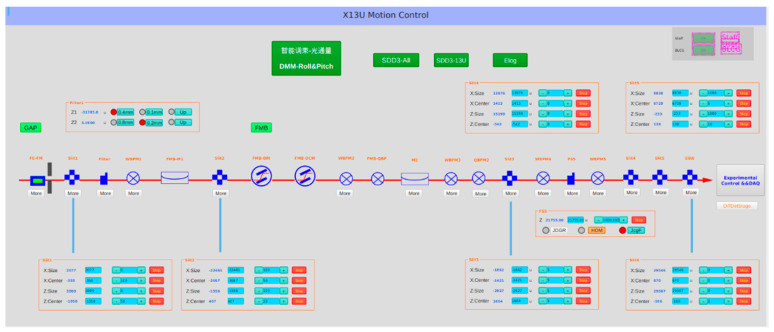
CSS-based OPI.

The EPICS control platform, integrated into the Linux system, offers a stable operating environment for intelligent beam optimization. The EPICS system incorporates various features such as remote equipment detection and control, automated script execution, closed-loop control, simulation modeling, visual data operations (acquisition, conversion, analysis, display, and storage), and security access mechanisms. These functionalities enable decentralized motion control and the centralized management of multiple motors along the beam line. EPICS showcases superior compatibility and expansibility, allowing it to seamlessly integrate with external programs. By utilizing the Pyepics module as a communication interface, a Python program for beam automatic optimization is developed to achieve intelligent control over the motor axes corresponding to the optical components. Furthermore, communication control between the Python program and MongoDB software is established through the Pymongo module. This allows for the retrieval and writing of expert experience to the database. The software structure design of the system is presented in Figure 6. The upper level Operation Interface (OPI) is developed based on CSS, as shown in Figure 7.

**Figure 8:**
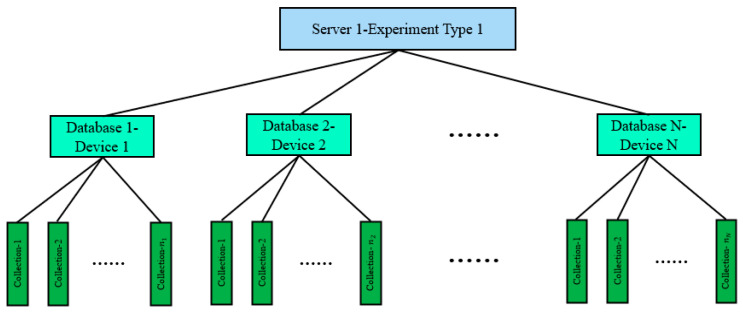
Database design structure, where the top layer is a server layer based on experimental types, the middle layer is a database layer based on optical devices, and the bottom layer is a collection layer based on motors.

Figure 8 depicts the database structure for BL13U intelligent beamline adjusting, designed using MongoDB software. As shown in Figure 8, the structure follows a top-down design scheme. The top layer represents the Server layer, which corresponds to one of the experiments conducted in BL13U. A dedicated server named “Server 1” is created for this experiment. The middle layer corresponds to the database layer, which encompasses the optical components used in the experiment. Each optical component serves as the basis for establishing a specialized database under the experiment type—optical components. The bottom layer represents the set layer, which corresponds to the motor motion axis included within the optical component. In this paper, sets are utilized as base units for storing all key–value pair character information within the experimental type—optical component—motor motion axis.

**Figure 9:**
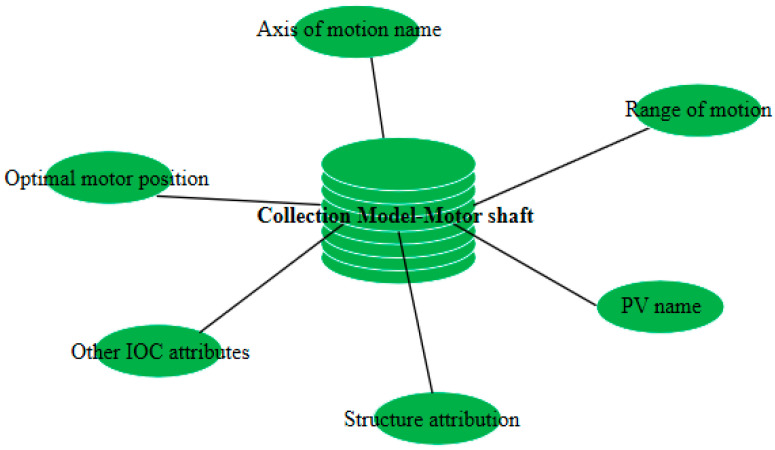
Ensemble model includes motor name, best position, range of motion, IOC attribute, etc.

The specific model of the set is shown in Figure 9. Each individual set stores information about a motor shaft, including its name, the PV connection name, the motion range (software limit), the directory it belongs to within the structure, the optimal position of the motor, and other IOC attributes. Among these attributes, the most crucial one is the information regarding the motor’s best position, which also gives the raw data that help the decision variable initialize.

## 3. Results

The system was deployed and tested at the SSRF hard X-ray nanoprobe beamline (BL13U). The synchronous light at the sample beamline must exhibit excellent performance, including strong flux and a stable spot position. Single-objective optimization tests only for flux and multi-objective optimization tests for both flux and spot position were conducted.

**Figure 10:**
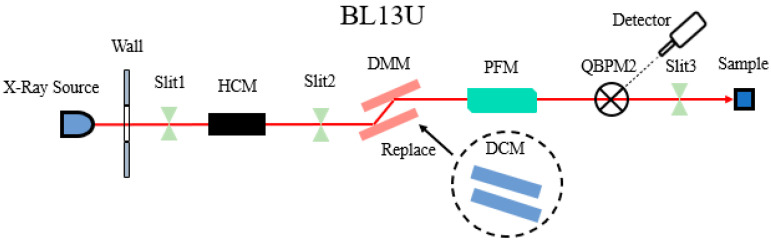
Layout diagram of Shanghai synchrotron radiation light source BL13U.

The BL13U beamline department houses important equipment, including the white slit, collimator (HCM, FMB, Oxford), multilayer monochromator (DCM, FMB, Oxford), pre-focusing mirror (PFM, Axilon, Germany), and light intensity detector (F2), along with other related auxiliary equipment. The optimization focused on the white slit Slit2, the multilayer monochromator DMM, and the pre-focusing mirror PFM. The beamline layout is illustrated in Figure 10.

SSRF advanced experiments demand the precise control of photon beam position stability. Therefore, the use of sensors to detect X-ray or photon beam position monitors (BPM) is very important. There are lots of different types of this kind of sensor, such as the following: strip-line monitor, blade beam position monitor (BBPM), fluorescent screen (FS), split plate ionization chambers, blade-type X-ray BMP (XBPM), quadrant PIN photodiode BPM (QBPM),wire scanning beam position monitor (WBPM), etc. [28,29,30,31,32,33].

For the single-objective optimization tests, the feedback parameter was maximum flux, which was the ultimate parameter for BL13U experiment. For the multi-objective optimization tests for both flux and spot position, the feedback parameters were maximum and spot position. For the parameter spot position, the distance between the center position of the spot and the position of the central light cone of QBPM was set as the optimization judgment; the smaller the distance, the better.

A quadrant beam position monitor (QBPM, FMB Oxford) consisting of four position-sensitive and flux-sensitive PIN diodes placed in an X-ray beam was adopted. The QBPM measured the incident X-ray flux and the beam position non-destructively.

Notably, the motor shaft of Slit2 has a wide range of motion, while those associated with DMM and PFM have a more limited range. The beam performance state at the secondary light source point was established using the four-way current signal from QBPM2 positioned behind the pre-focusing mirror. The motor shafts exhibiting noticeable optical attitude changes in the equipment were chosen for optimization. Table 1 illustrates the measured motor shafts and their respective physical significance.

**Figure 11:**
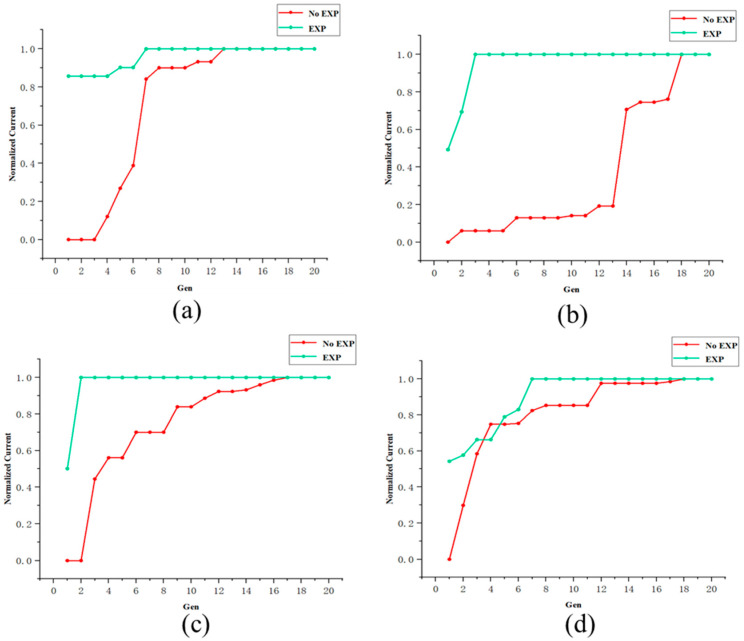
Optimization process of four groups of online tests. The horizontal axis represents the current evolutionary iteration, and the vertical axis represents the current intensity after normalization. The red curve represents the results under the random initial state, while the green curve represents the results when invoking the prior experience. (**a**) The optimized objects are Slit2-X1, X2, Z1, Z2. (**b**) The optimized objects are DMM-Roll. (**c**) The optimized objects are DMM-Pitch, Roll-, PFM-m1, and m2. (**d**) The optimized objects are DMM-Pitch and Roll.

Two control tests were conducted for the same group of motor shafts. The first test involved a random initial state, while the second test utilized beam adjusting experience for initialization. The experience of BL13U beam optimization during the simulation stage showed that using a population size of 10 and an evolution number of 20 yielded better results. Therefore, these parameter settings were adopted for the tests. Table 2 presents the results of the single-objective test based on flux, comprising eight rounds of tests across four groups. The optimization process of the test is illustrated in Figure 11, where the horizontal axis represents the evolution iteration and the vertical axis represents the flux value of the best individual in the population at each iteration.

**Figure 12:**
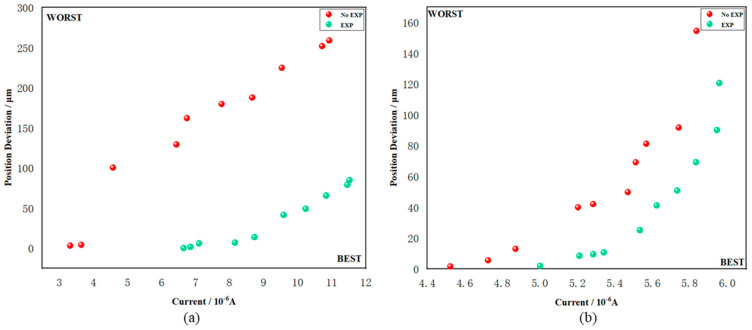
Optimization process of four groups of online testing. The horizontal axis represents the flux intensity, while the vertical axis represents the degree of deviation between the spot position and the central light cone. Each point in the figure represents an equivalent Pareto solution, with the red point indicating the result under the random initial state, and the green point representing the result under experience. (**a**) The optimized objects are Slit2-X1, X2, Z1, and Z2. (**b**) The optimized objects are DMM-Pitch and Roll.

Results related to the dual objectives (flux and spot position tests) are shown in Table 3, with a total of two groups tested. Figure 12 represents the visual test results, where the horizontal axis indicates the flux value, and the vertical axis depicts the degree of spot deviation.

## 4. Discussion

The results of the single-objective tests indicate the following:

(1) The system can optimize the device to converge under the current beam state, enabling the acquisition and storage of the best optical attitude information;
(2) Apart from the algorithm’s own parameter settings, the speed of system optimization primarily relies on the number of optimized motor shafts and the travel range associated with each shaft. The more motors there are and the wider their travel range, the longer it takes to achieve convergence. The latter factor plays a decisive role. In the same difference operator experiment, the subsequent operator experiment only begins when all motors have reached the specified position;
(3) When considering the slightly different beam states before and after conducting the same set of experiments, invoking the database experience yields significantly improved convergence rates in comparison to optimization results obtained under the initial random condition. This improvement demonstrates a significantly faster convergence speed, with the speed increasing 15 times;
(4) At the start of the system’s optimization, the evolutionary range exhibits significant changes. However, as the system gradually approaches the optimal solution, the rate at which the evolutionary range decreases gradually slows until convergence.

The results of the dual-objective tests indicate the following:

(1) During the optimization process, the algorithm iterates comprehensively towards two optimization objectives. Under the current beam state, the flux is optimized towards the maximum objective, while the spot position deviation is optimized towards the minimum objective. However, actual test results demonstrate that the two optimization paths are not entirely aligned and may even diverge. In such cases, the location of the obtained Pareto equivalent solution on the two-dimensional plane represents the algorithm’s comprehensive optimization results based on both objectives. These positions are represented by individual Pareto points in Figure 11. The ordinate represents the position deviation (measured in microns), and the abscissa represents the current intensity (measured in microamperes);
(2) When optimizing from a random initial state, the resulting Pareto equivalent solutions tend to be scattered and irregularly distributed near the Pareto frontier curve. This indicates that the algorithm exhibits weak non-dominance based on both objectives within a finite number of iterations, and there still remains some distance from the ideal convergence state. On the other hand, when invoking the expert system’s beam adjusting experience for optimization, the evolved Pareto frontier points are distributed along a smoother curve, forming a more regular solution boundary. This suggests that the algorithm achieves better non-dominance based on the finite evolution of the both objectives, and has approached or even reached the ideal convergence state;
(3) The optimization results for a single objective are superior to those obtained when optimizing both objectives simultaneously. However, it should be noted that since the beam is currently undergoing debugging, this variation is mainly attributed to the challenge of stabilizing the spot itself. Despite the empirical results not being dominant in terms of optimization time, the obtained solution set surpasses that of the random initial state. Overall, the benefits and improvements achieved in solving nonlinear problems with multiple objectives are more significant.

## 5. Conclusions

An intelligent control system for beam optimization based on the hardware and software characteristics of the hard X-ray nanoprobe beamline has been designed, developed, and successfully applied to the hard X-ray nanoprobe beamline at Shanghai Synchrotron Radiation Facility. This system utilizes a global automatic evolution algorithm and an expert system. The global automatic evolution algorithm is implemented using modular packaging and nested calls in Python language. The expert system is implemented through core software such as MongoDB and CSS. It takes feedback signals, including the flux and spot position of the secondary light source point of the beamline, to achieve the iterative optimization of the beamline equipment based on the EPICS system. The system achieves the optimal beam state in terms of optical performance. These beam adjusting results, containing motor position information, are stored in the expert system’s database as valuable experience values for future beam adjusting iterations. This feature enables the adaptive learning function of the system. The test results demonstrate that the intelligent control system can accurately optimize the device to achieve the optimal optical attitude under different beam conditions. The maximum optimization time does not exceed 9 min.

The test results provide detailed insights, revealing that introducing beam adjusting experience from the expert system can increase the convergence rate by 15 times in single-objective optimization, and lead to a better solution set in both-objective optimization. The testing also shows that the intelligent optimization-based beam adjustment method not only achieves global automatic optimization, surpassing the efficiency of manual beam adjustment, but also continuously improves the quality of experience values through adaptive learning, enabling self-improvement of the system. However, it is worth noting that most of the optimization time is consumed by hardware response and motion. As equipment increases and factors such as poor motion sensitivity and power affect the motor’s performance, the optimization time can significantly increase. Therefore, upgrading and analyzing the hardware link becomes crucial. This study integrates the expert system into intelligent beam modulation for the first time, enhancing the effect of intelligent beam modulation for the complex beamline at SSRF. This provides valuable insights into beam optimization ideas for the other beamlines at SSRF, and other synchrotron radiation devices.

At present, the intelligent control system used for beamline optimization based on the automatic evolution algorithm and expert system has begun to be applied online at BL13U. In the later stage, automatic beamline optimization and data acquisition will be combined. During the experiment, the quality of the X-ray will be monitored while the experimental data are being collected, and the beam will be automatically optimized so as to obtain higher-quality data, and to derive a more efficient and more automatic experimental process.

## Figures and Tables

**Table 1:** Physical significance of motor shaft movement of the equipment under test.

Optical	Motors	Physical Function
Slit2	X1,X2 Z1,Z2	Adjust the knife size of the through light in the X and Z directions
DMM	Pitch	Adjust the pitch angle of 2nd crystal
	Roll	Adjust the roll angle of 2nd crystal
PFM	m1,m2	Adjust the pitch angle of the mirror

**Table 2:** Conditions and results of single objective test.

Order	Optimized Device	Initial State	Convergent Algebra	Convergence Time (S)
1	Slit2 (X1,X2,Z1,Z2)	Random	13	234
2	Slit2 (X1,X2,Z1,Z2)	Experience	7	124
3	DMM (Roll)	Random	18	58
4	DMM (Roll)	Experience	3	9
5	DMM (Pitch,Roll), PFM (m1,m2)	Random	17	182
6	DMM (Pitch,Roll), PFM (m1,m2)	Experience	1	6
7	DMM (Pitch,Roll)	Random	18	105
8	DMM (Pitch,Roll)	Experience	7	47

**Table 3:** Conditions and results of dual-objective tests.

Order	Optimized Device	Initial State	Optimization Time (S)	Position Deviation (μm)	Flux (10^−6^ A)
1	Slit2 (X1,X2,Z1,Z2)	Random	506	150.18	7.23
2	Slit2 (X1,X2,Z1,Z2)	Experience	530	34.81	9.11
3	DMM (Pitch,Roll)	Random	135	54.90	5.27
4	DMM (Pitch,Roll)	Experience	179	42.80	5.55

## Data Availability

The data presented in this study are available on request from the corresponding author.
